# The Comparison of Three Statistical Models for Syndromic Surveillance in Cattle Using Milk Production Data

**DOI:** 10.3389/fvets.2020.00067

**Published:** 2020-03-06

**Authors:** Anouk M. B. Veldhuis, Wim A. J. M. Swart, Henriëtte Brouwer-Middelesch, Jan A. Stegeman, Maria H. Mars, Gerdien van Schaik

**Affiliations:** ^1^Royal GD, Deventer, Netherlands; ^2^Department of Farm Animal Health, Faculty of Veterinary Medicine, Utrecht University, Utrecht, Netherlands

**Keywords:** veterinary syndromic surveillance, aberration detection methods, vector-borne diseases, cattle, milk production data

## Abstract

Two vector-borne infections have emerged and spread throughout the north-western part of Europe in the last decade: Bluetongue virus serotype-8 (BTV-8) and the Schmallenberg virus (SBV). The objective of the current study was to compare three statistical methods when applied in a syndromic surveillance context for the early detection of emerging diseases in cattle in the Netherlands. Since BTV-8 and SBV both have a negative effect on milk production in dairy cattle, routinely collected bulk milk recordings were used to compare the three statistical methods in their potential to detect drops in milk production during a period of seven years in which BTV-8 and SBV emerged. A Cusum algorithm, Bayesian disease mapping model, and spatiotemporal cluster analysis using the space-time scan statistic were performed and their performance in terms of sensitivity and specificity was compared. Spatiotemporal cluster analysis performed best for early detection of SBV in cattle in the Netherlands with a relative sensitivity of 71% compared to clinical surveillance and 100% specificity in a year without major disease outbreaks. Sensitivity to detect BTV-8 was low for all methods. However, many alerts of reduced milk production were generated several weeks before the week in which first clinical suspicions were reported. It cannot be excluded that these alerts represent the actual first signs of BTV-8 infections in cattle in the Netherlands thus leading to an underestimation of the sensitivity of the syndromic surveillance methods relative to the clinical surveillance in place.

## Introduction

Syndromic surveillance aims at identifying unusual increases in health related events in a population based on data aggregated across the monitored population and has been used frequently in the field of public health surveillance. The field of veterinary syndromic surveillance focusses on detection of emerging diseases as well as changes in trends of endemic diseases. It has been rising during the last decade due to increasing availability of relevant data sources and interest of veterinary epidemiologists for syndromic surveillance (1, 2). Often, the first step in the detection process is the construction of temporal time series of the data that are being monitored (cases, rates, counts, etc.), to define a baseline model for the expected number of events. The next step is the application of statistical methods to distinguish if an observed event rate is significantly different from the expected levels defined by the historical baseline. In these analyses, the modeling approach can be directed at temporal abnormalities, spatial abnormalities or a combination of both (3).

In the temporal context, time series from a region or country as a whole are inspected prospectively for abnormalities, for example in the form of statistical process control charts. Statistical process control charts are quality control methods used to monitor production processes over time to detect changes in process performance (4). Various statistical process charts have been developed since early in the twentieth century. The cumulative sum (Cusum) chart is an example of a method that is known for its ability to detect small shifts in the parameter of interest (5). The principle of the Cusum algorithm in its simplest form is that it accumulates deviations between expected and observed values (e.g., counts, percentages, rates, etc.). Generally, an arbitrary constant *k* is chosen to explain the variation of the mean of the baseline period (meaning that deviations smaller than *k* will not be summed). If the process remains “in control” the cumulative sum should fluctuate stochastically around zero. Identification of aberrations occurs when the absolute cumulative sum of differences between expected and observed values exceeds a fixed control limit (*h*) to be chosen by the user.

Alternatively, when focusing on detecting abnormalities in a spatial context in the data source, statistical methods are aimed at detecting regions in which the distribution of the parameter of interest within a certain timeframe is abnormally high (or low) compared to other regions. This is known as disease mapping, aiming at quantifying the amount of true spatial heterogeneity and its associated patterns to highlight areas of elevated risk (6). Disease mapping methods vary broadly from being non-parametric in nature (e.g., smoothing models) to more complex Bayesian random effects models (7). Bayesian disease mapping models treat the relative risks as random variables and specify a distribution for them, to capture the unexplained variability in the observed data that might be the result of (local) disease outbreaks. In the 1990s, Besag et al. (8) proposed to split the relative risk parameter in separate variance components, i.e., spatially uncorrelated variance heterogeneity and spatially correlated heterogeneity. This led to the introduction of Bayesian disease mapping models based on conditional autoregression (CAR), i.e., the value for any given spatial unit is estimated conditionally on the values of neighboring units. In these models, the estimated correlation structure between neighboring spatial units is used as prior distribution for the (correlated) spatial random effect. This prior distribution is then combined with the likelihood of the observed data to obtain a posterior distribution for the random effect(s), followed by inference to identify areas where the relative risk exceeds a predefined value (9).

The most widely used method in the field of spatiotemporal modeling is the space-time scan statistic as proposed by Kulldorff (10). With this method, a cylindrical window scans across all units in space and time in the data source, noting the number or value of observed and expected observations inside the window. In a prospective analysis only those cylinders that include the end of the study period are considered, hence excluding clusters from the past. A likelihood ratio statistic is then computed for each space-time window, by comparing the likelihood of the observed data (given the value or rate of events within and outside the window) and the likelihood function assuming the rate of events within and outside the window are equal (10, 11). The method adjusts for multiple testing by evaluating only the significance of the window with the maximum likelihood ratio statistic over all cylinders (i.e., the most likely cluster), using a *p-*value obtained from Monte Carlo simulations (12).

Two vector-borne infections have emerged and spread throughout the north-western part of Europe in the last decade: Bluetongue virus serotype-8 (BTV-8) and the Schmallenberg virus (SBV). BTV-8 emerged in August 2006 and re-emerged in July 2007 and caused important economic losses (13). In the outbreak of 2007, the most prominent clinical signs observed in affected cattle farms were fever, lameness/stiffness, conjunctivitis, nasal discharge, crusts/lesions of nose and/or mouth, redness/lesions of teats and a drop in milk yield (14). SBV emerged in north-western Europe in the late summer of 2011, causing diarrhea and drop in milk production in adult cattle (15) and congenital malformations in new-born ruminants (16). Since BTV-8 and SBV both have a negative effect on milk production in dairy cattle, routinely collected milk production records may have the potential to be used for early-warning syndromic surveillance of such pathogens. In a previous study, we assessed the value of routinely collected milk production data for the early detection of emerging vector-borne diseases in cattle using the space-time scan statistic (17). Although the results seemed promising, a number of improvements were suggested for future use of such data. For example, milk yield was calculated based on monthly “test-day” milk recording data, providing monthly observations on herd level. As an alternative, the use of bulk milk collection data (resulting in approximately three observations per herd per week) might increase timeliness and sensitivity of detection of disease outbreaks. Also, milk yield was analyzed with a single statistical method. The objective of the current study was to compare three statistical methods, the Cusum chart, Bayesian disease mapping and prospective spatiotemporal cluster analysis, to assess their potential to detect drops in milk yield during a period of seven years in which BTV-8 and SBV emerged using routinely collected bulk milk collection data.

## Materials and Methods

### Data

**Figure 1:**
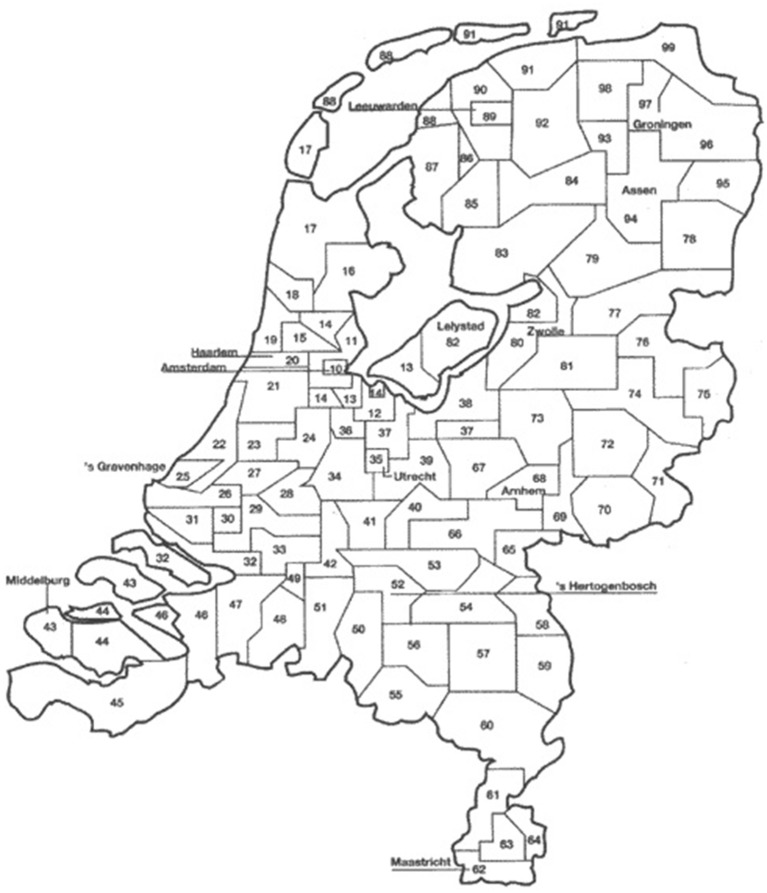
Two-digit postal districts in the Netherlands (*N* = 90).

A total of 13,330,908 bulk milk collection recordings (2–3 records per week per herd) from 21,074 dairy farms (~96% of the Dutch dairy herds) were obtained between July 1, 2005 and December 31, 2011. These data were provided by seven dairy processors. Herd size information on monthly level was obtained from the national identification and registration (I&R) database and was used to estimate the number of lactating cows per herd per day. It was assumed that at any time point, 10% of the cows >2 years of age in a herd were in the dry period and therefore not taken into account in the number of lactating cows per herd. For each herd, the average milk yield per animal per day was calculated by dividing the total amount of collected bulk milk on a given day (taking into account the number of milkings since the last collection) by the estimated number of lactating cows. Days with an average milk production >50 kg/cow were considered administrative errors and excluded prior to analysis (1% of the data). Herd location data on postal district level (2-digit postal code) was obtained from Royal GD. The 90 postal districts of the Netherlands are shown in Figure 1. The mean number of herds per postal district decreased from 221 in 2007 to 195 in 2011. Bulk milk data from 2005 to 2006 were incomplete for 37% of the 90 postal districts (mainly in the northern and eastern area of the country; districts 65 to 99), due to a merger of the largest dairy processor in the Netherlands in 2007. This part of the Netherlands was not affected yet by BTV-8 in 2006 (18).

### Data Analysis

#### Construction of the Baseline Model

Time series analyses were carried using STATA/SE version 14 software (19). Due to the large amount of milk production records and to guarantee the privacy of herds, aggregation of the milk yield per cow per herd by calculating its mean at postal district-week level was done prior to statistical analyses. Time series of the mean milk yield per cow per postal district per week was constructed using a harmonic linear regression model (regress) (Equation 1). Annual seasonality was taken into account by including two sine/cosine harmonics as predictors. The number of harmonics, n, was chosen according to the AIC criterion to best fit the observed milk production data.

(1) $$\begin{array}{l} y_{ij}=\beta_{0}+\beta_{1}district_{i}+\;\beta_{2}week_{j}+(\sum_{n=1}^{2}\alpha_{n}\times \cos(\frac{2\pi^{*}t^{*}n}{52})\\ \;+\;\beta_{n}\times \sin(\frac{2\pi^{*}t^{*}n}{52}))+\;\varepsilon_{ij} \end{array}$$

where:

*E*(*y*_*ij*_) = the expected milk yield per cow in postal district *i* in week *j*
β_0_ = intercept (constant)
α_*n*_, β_*n*_ = phase and amplitude parameters
*t* = the number of weeks since the first week in the dataset
*n* = harmonic number
ε_*ij*_ = random error for district *i* in week *j*.

The time series model was run in a time-periodic prospective fashion, i.e., repeating the analysis every week with an updated (moving) baseline period. In each model-run, the expected milk yield per cow in postal district *i* in week *j* was predicted using up to two preceding years (104 weeks) as baseline. By doing so, we simulated the use of the system as if it were applied in real-time on a weekly basis, i.e., on a practically feasible manner. These time-periodic analyses were carried out for three separate time periods (Table 1).

**Table 1:** Boundaries of the baseline and prediction periods for the construction of time series and corresponding week-by-week prediction of mean milk yield per cow in the Netherlands, including the mean root mean squared error of the model (RMSE) calculated over the baseline period (and its standard deviation).

**Model**	**Baseline period**	**Prediction period**	**Mean RMSE (sd)**
BTV-model	July 1, 2005–June, 30, 2006	July 1, 2006–Dec. 31, 2007 (79 weeks)	0.74 (0.06)
SBV-model	Jan. 1, 2009–Dec. 31, 2010	Jan. 1, 2011–Dec. 31, 2011 (52 weeks)	0.58 (0.02)
Control-model	Jan. 1, 2008–Dec. 31, 2009	Jan. 1, 2010–Dec. 31, 2010 (52 weeks)	0.66 (0.04)

In the first model (“BTV-model”), the expected milk yield was estimated for each postal district from week 27 of 2006 (i.e., the first week of July) to week 52 of 2007. It was hypothesized that during parts of this period, milk production was affected as a result of the BTV-8 epidemic, which started in the Netherlands in the summer of 2006 (20) and re-emerged (more intensively) in the summer of 2007 (21). The BTV-model used only data from postal districts for which complete time series could be constructed, thus excluding the northern and eastern areas for which data from 2005 to 2006 were incomplete or missing (*N* = 35). The expected milk yield in the first predicted week (week 27 of 2006) was estimated using the preceding 52 weeks as baseline. After the first predicted week, the baseline was extended with extra week(s) to a maximum of 104 weeks up to the prediction of week 27 of 2007. For the weeks thereafter, the expected milk yield for each week was predicted using a baseline period of 104 weeks. A second model was used to investigate drops in milk production as a result of the SBV epidemic, which started in the Netherlands in the later summer of 2011 (15, 22). In this model (“SBV-model”), the expected milk yield was estimated for each postal district-week from week 1 to 52 of 2011, using data from the two preceding years (104 weeks of 2009–2010) as baseline. A third model was used as control model (“Control-model”) in which drops in milk production in 2010 were investigated, a year without major epidemics in the Netherlands. The expected milk yield was estimated for each postal district-week from week 1 to week 52 of 2010, using data from the preceding 2 years (2008–2009) as baseline.

The root mean squared error (RMSE) of each week-run was calculated and averaged per model as a measure of fit of the time series models over the baseline period (Table 1). RMSE is a common metric used to measure accuracy for continuous variables. The observed and predicted milk yield estimates in each district-week were obtained after each run of each model. These were subsequently used for further analyses using a Cusum algorithm, a Bayesian disease mapping model and a prospective spatiotemporal scan statistic.

#### Cusum Analysis

Cusum analyses were carried out per postal district after each run of the moving time series analysis. Aiming to detect drops in milk production, a one-sided negative Cusum function was used to calculate the cumulative sum of differences between observed and predicted milk production (Equation 2), inspired by Lawson and Kleinman (9) and Marceau et al. (23). The algorithm that was applied to the time series of each district *i* can be described as follows:

(2) $$\begin{array}{l} Cusum_{t}=\min\left\{0,Cusum_{t-1}+\left(y_{t}-{\hat{y}}_{t}-k_{i}\right)\right\} \end{array}$$

where *t* is the time unit in weeks, *y*_*t*_ is the observed milk yield in district *i* at week *t*, ŷ_*t*_ is the predicted milk yield in district *i* at week t and *k*_*i*_ is a reference value to explain the variation of the mean of the baseline period for district *i*. For *k*, the 5th and 10th percentile of the difference between observed and predicted milk yield estimates (the residual) from the baseline period within district *i* was used. As these residuals fluctuate around 0, the 5th and 10th percentile of the distribution of the residuals (and thus *k*) have a negative value. Please note that a negative Cusum function was used, shown by the “min” in Equation 2, as we are interested in detecting drops in milk production, i.e., observed minus predicted milk yield being <0. The algorithm works as follows. At t_0_, the Cusum is set at 0. Once *y*_*t*_ − ŷ_*t*_ (the residual) is negative and more extreme than *k*, the Cusum value is changed to (*y*_*t*_ − ŷ_*t*_ − *k*_*i*_). If the residual is less extreme the next weeks, by being less negative than *k* or even positive, the Cusum value decreases and will reach 0 once (*y*_*t*_ − ŷ_*t*_ − *k*_*i*_) >0. By doing so, a series of Cusum-values (the “Cusum chart”) is created for each district. The control limit *h*_*i*_ was then applied to the Cusum chart. The first week in which the Cusum value is more extreme than *h* was considered the alert week. Only alerts generated in the prediction week were kept for interpretation, i.e., alerts from the past were considered meaningless. To find the optimal balance between the algorithm's timeliness, sensitivity and specificity, a number of values of *h*_*i*_ were explored as a function of *k*_*i*_: 1.5*k*, 2*k*, 2.5*k*, and 3*k*. A threshold value of 2.5^*^k_*i*_ yielded best results and was therefore used as control limit *h*_*i*_. A graphical example of a Cusum chart can be found in Supplementary Figure 1.

#### Bayesian Disease Mapping Analysis

Spatial disease mapping models typically assess the spatial association of case event or count data [reviewed by (9)], expressing localized variation in disease risk as relative risks. We constructed a disease mapping model that estimates residuals instead of relative risks, by adding a spatial component to the residuals per district of the baseline model (Equation 1). A purely spatial model was developed using OpenBUGS (24) to identify areas in which the probability of a lower than expected milk production exceeds a predefined threshold (Equation 3), inspired by the conditional autoregression (CAR) models described by Richardson et al. (25) and Lawson (26). The model can be described as follows:

(3) $$\begin{array}{l} \;sres_{i}=\;\frac{({\text{y}}_{\text{i}}-\;{\hat{y}}_{i})+\;{\text{u}}_{\text{i}}}{\sigma_{\text{i}}\;/\;\sqrt{{\text{nherd}}_{\text{i}}}}=\text{standardized residual }\\ \text{ for district }i\\ \;y_{i\;}=\text{observed milk production in district }i\\ \;{\hat{y}}_{i}=\text{expected milk production in postal district}\\ \;i,\text{ based on the time series regression }\\ \text{ model }(\text{Equation }1)\\ \;u_{i}\;\sim \;N(b_{i},\;\tau n_{i})\;=\text{spatial random effect for district }i\text{ where }\\ \;\tau \text{ is the precision weighted by }{\text{n}}_{\text{i}},\text{ i}.\text{e}.,\\ \text{ the number of neighboring districts}\\ \text{ of district }i\;{\text{and b}}_{\text{i}}\text{ is the mean of the spatial}\\ \text{ component in the set of districts adjacent to}\\ \text{ district }i \end{array}$$

The model was run time-periodically, using only observed (*y*_*i*_) and predicted (ŷ_*i*_) milk yield data from week *j* per run. In the model, a spatially correlated random effect (*u*_*i*_) was added to the residual milk yield estimates (*y*_*i*_ − ŷ_*i*_) derived from the time series regression model (Equation 1), creating a new residual. The model included a correlation structure between postal districts by specifying a weighted conditional autoregressive Gaussian distribution (CAR) as prior for the correlated spatial random effect (*u*_*i*_).The new model residuals were standardized (*sres*_*i*_) by adjusting them to the number of herds per district *i* (*nherd*_*i*_). Non-informative prior distributions were given to the precision τ of the spatial random effect [gamma(0.001,0.001)]. Residual thresholds of −1 and −5 were used to calculate the posterior exceedance probabilities for each district *i*. Alerts were defined as district-week combinations where P(*sres*_*i*_ < −5) or P(*sres*_*i*_ < −1) was more than 0.99. The model was compiled with two sets of initial values. A burn-in period of 5,000 iterations was applied; conclusions are based on the next 10,000 iterations. The Brooks-Gelman-Rubin diagnostic was used in randomly chosen model runs to assure that the two chains had converged (27), inspecting the plots for the potential scale reduction factor being very close to 1. Geweke's test was used in randomly chosen model runs to detect signs of failing convergence (28).

#### Spatiotemporal Cluster Analysis

Model residuals at postal district-week level, comprising the complete baseline period plus the predicted week, were uploaded in SaTScan™ (29) after each model run to identify space-time clusters of low milk production. Prospective analyses were carried out using the normal probability model in SaTScan™. Model residuals were weighted by the square root of the number of herds per district-week to account for uncertainty in residuals from areas with a low cattle herd density. A circular window shape was chosen. We scanned for “low mean” clusters, i.e., lower observed milk production than would have been expected. For each window, a likelihood-ratio test statistic was calculated and the window with the maximum value was considered the cluster that is least likely to have occurred by chance. Its distribution under the null hypothesis and its corresponding *p*-value was obtained by Monte Carlo hypothesis testing (999 simulated random datasets). Clusters can comprise multiple districts. The maximum spatial cluster size was set at 5 and 10% of the population at risk. These relatively low cluster sizes were chosen as the spatial scan statistic was used for early detection of clusters of reduced milk production. The maximum temporal cluster size was set at 1 week. Clusters of low milk production were defined as windows with a *p* ≤0.01.

### Evaluation of Performance

To compare and evaluate the performance of the different statistical methods, sensitivity and specificity of detecting the BTV-8 and SBV epidemics were calculated during corresponding time periods for each method. A dataset with suspicions of clinical BTV-8 infections in cattle in 2006 and 2007 was obtained from the Netherlands Food and Consumer Product Safety Authority, from which the first confirmed suspicion per district was derived (referred to as “BTV-reference signals”). It must be noted that BTV-8 indicative clinical signs in affected animals were more prominent in sheep flocks than in cattle farms. Also, according to Elbers et al. (30), during the BTV-8 outbreak in 2006 clinical signs had started ~12–17 days before a suspicion was reported to the veterinary authorities. In the Netherlands, notifying SBV suspicions was not mandatory up to December 2011. An overview of clinical SBV suspicions in adult cattle, notified voluntarily by farmers and veterinarians in August and September 2011 (when the existence of SBV was still unknown), was obtained from Royal GD. The notifications were based on a sudden drop in milk production, diarrhea and/or fever (15). From the list of notifications, the first suspicion per district was derived (referred to as “SBV-reference signals”). With these two sets of reference signals, sensitivity of detection by the statistical methods—relative to the clinical surveillance in place—was calculated as the proportion of first suspicions that was preceded by a statistical alert. Due to the delay between infection and appearance of clinical signs, mildness or absence of clinical signs in cattle, and limited awareness of BTV-8/SBV in the initial stage of the epidemic, it was assumed that reporting of clinical suspicions were probably somewhat delayed in terms of representing the first moment of infection in a district. Therefore, alerts were considered true when the alert was generated in the week of the suspicion or in the 4 weeks prior to the suspicion. A sequence of true alerts within one district was counted as one true alert. Alerts in the weeks after the first clinical suspicion in a district were ignored. By doing so, sensitivity was adjusted for timeliness. To measure the effectiveness of each method per year, a predictive alert value was calculated as the proportion of alerts per year that were considered to be true. This is indicative for the amount of false alerts per method. Specificity was calculated per district as the proportion of weeks for which no alert was generated in 2010, i.e., the year without major disease outbreaks. Specificity values of the 90 districts were averaged to obtain an overall estimate of specificity per method.

## Results

**Figure 2:**
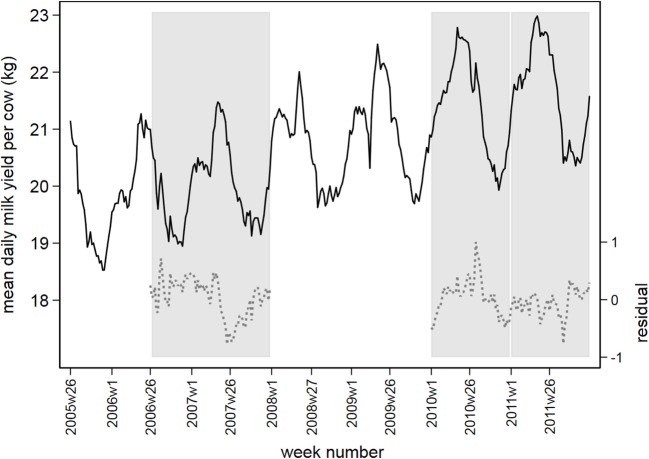
Observed daily milk yield per cow (black solid line) and residual values following time series analysis (gray dashed line), averaged by week between July 1, 2005 and December 31, 2011. Prediction periods in 2006/2007, 2010, and 2011 are marked in vertical gray bars.

Figure 2 displays the observed and residual daily milk yield averaged by week over all postal districts, with the three prediction periods marked in gray. The mean residual daily milk yield fluctuates around 0 between −1 and +1 in the prediction periods.

### Sensitivity and Predictive Alert Value

**Figure 3:**
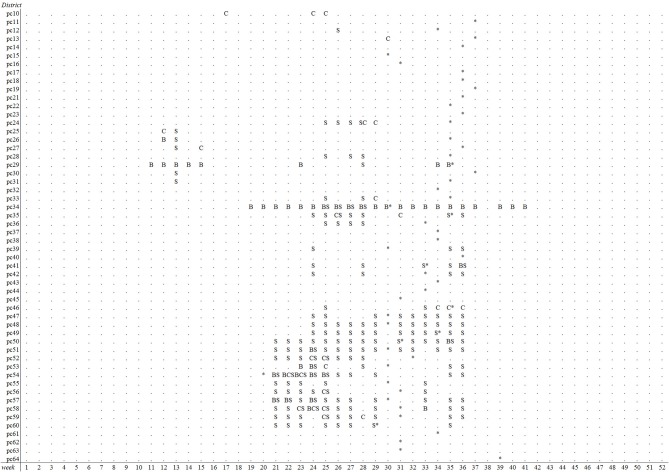
Overview of postal districts and weeks with alerts of reduced milk production in week 1–52 of 2007, based on prospective weekly Cusum analysis (C) with *k* = P10, spatiotemporal cluster analysis (S) with a maximal spatial window of 10% and Bayesian disease mapping analysis (B) using a residual threshold of −5. First BTV-8 confirmed suspicions in cattle per district are indicated with an asterisk (districts 65 to 99 were omitted).

**Figure 4:**
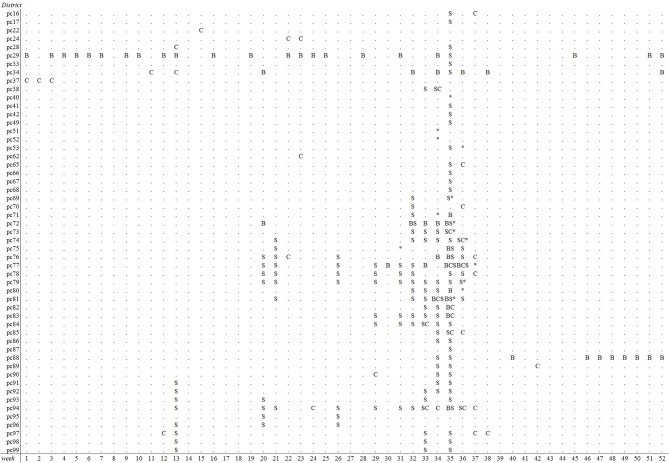
Overview of postal districts and weeks with alerts of reduced milk production in week 1–52 of 2011 based on prospective weekly Cusum analysis (C) with *k* = P10, spatiotemporal cluster analysis (S) with a maximal spatial window of 10% and Bayesian disease mapping analysis (B) using a residual threshold of −5. The week and location of first SBV suspicions is indicated with asterisks.

The emergence of BTV-8 in 2006 could only be detected by the Bayesian disease mapping method with setting “sres < −1”. A total of 54 alerts were generated in 2006 by this method, of which two were prior to the 29 BTV-reference signals that year, leading to a sensitivity value of 6.9% (Table 2). Irrespective of the parameter settings used, the other methods failed to detect the emergence of BTV-8 in 2006 earlier than the passive clinical surveillance that was in place (Table 2). The spatiotemporal cluster analysis method generated some alerts in districts with clinical suspicions in cattle, yet up to 9–10 weeks prior to the BTV-reference signals. Multiple alerts were generated by each method for 2007, except with the Cusum algorithm using the 5th percentile (P5) as *k* (Table 2). A graphical overview of the alerts per model and district-week in 2007 is illustrated in Figure 3. Each method generated alerts for districts with clinical suspicions in cattle, up to 23 weeks prior to the BTV- reference signals (Figure 3). Sensitivity values for 2007 were therefore low and ranged from 1.2% for the Cusum algorithm with setting “*k* = P10” to 22.1% for Bayesian disease mapping with setting “sres < −1.” Spatiotemporal cluster analyses, with parameter setting “spatial 10%,” however yielded a predictive alert value of 39%, indicating that 39% of the 36 alerts generated in 2007 were considered true alerts. For 2011, each method generated multiple alerts, of which the majority are clustered around week 32–37 (Figure 4). Sensitivity values for 2011 ranged from 7.1% for the Cusum algorithm with setting “*k* = P5” to 78.6% for Bayesian disease mapping with setting “sres < −1.” Predictive alert values however appeared highest for spatiotemporal cluster analyses, with 64.3% for the model with setting “spatial 5%” and 55.6% for the model with setting “spatial 10%.” In general, Bayesian disease mapping, irrespective of parameter setting, produced lowest predictive alert values due to the large number of alerts relative to the number of true alerts.

**Table 2:** Total number of alerts per year (N) and performance metrics per method for the BTV-model (2006 and 2007), the SBV-model (2011) and the NC-model (2010).

	**BTV-model**						**SBV-model**			**Control-model**	
	**2006**			**2007**			**2011**			**2010**	
Method^*^	***N***	**SE (95% C.I.)**	**PV (95% C.I.)**	***N*^#^**	**SE (95% C.I.)**	**PV (95% C.I.)**	***N*^#^**	**SE (95% C.I.)**	**PV (95% C.I.)**	***N*^#^**	**SP (sem)**
**Cusum**											
*k* = P5	0	0.0% (0.0–11.9)	–	0	0.0% (0.0–4.2)	–	12	7.1% (0.2–33.9)	10.8% (3.0–25.4)	0	100% (0.00)
*k* = P10	0	0.0% (0.0–11.9)	–	25	1.2% (0.0–6.3)	4.0% (0.1–20.4)	37	28.6% (8.4–58.1)	8.3% (0.2–38.5)	8	99.8% (0.09)
**STCA**											
Spatial 5%	2	0.0% (0.0–11.9)	0.0% (0.0–84.2)	13	1.2% (0.0–6.3)	7.7% (0.2–36.0)	14	64.3% (35.1–87.2)	64.3% (35.1–87.2)	0	100% (0.00)
Spatial 10%	3	0.0% (0.0–11.9)	0.0% (0.0–70.8)	36	16.3% (9.2–25.8)	38.9% (23.4–56.5)	18	71.4% (41.9–91.6)	55.6% (30.8–78.5)	0	100% (0.00)
**BDM**											
sres < −5	5	0.0% (0.0–11.9)	0.0% (0.0–52.2)	46	2.3% (0.3–8.1%)	4.3% (0.5–14.8)	55	28.6% (8.4–58.1)	7.3% (2.0–17.6)	26	99.4 (0.33)
sres < −1	54	6.9% (0.1–22.8)	3.7% (0.5–12.7)	458	22.1% (13.9–32.3)	4.1% (2.5–6.4)	598	78.6% (49.2–95.3)	1.8% (0.9–3.3)	489	89.6 (1.21)

*Sensitivity (SE) and predictive alert value (PV) are expressed as a percentage with 95% confidence interval (95% C.I.). Specificity is expressed as mean percentage with standard error of the mean (sem)*.

* *Cusum with k being the 5th (P5) or 10th (P10) percentile value of residuals; Spatiotemporal cluster analysis (STCA) with a maximal spatial window of 5% or 10%; Bayesian disease mapping (BDM) with a standardized residual (sres) threshold of −1 or −5*.

# *With regard to STCA, each cluster is counted as one alert in the total number of alerts per year, irrespective of the number of districts included per cluster*.

### Specificity

In 2010, zero alerts were generated with the spatiotemporal cluster analysis, irrespective of parameter setting, resulting in a specificity estimate of 100% for this method (Table 2). Eight alerts were generated by the Cusum algorithm with setting “*k* = P10,” all in week 5–9, resulting in a mean specificity estimate of 99.8%. Bayesian disease mapping analysis with setting “sres < −5” yielded 26 alerts, corresponding to a mean specificity of 99.4%. Most alerts were generated with Bayesian disease mapping analysis with setting “sres < −1,” resulting in a mean specificity of 89.6%.

## Discussion

Recent examples in the field of cattle health surveillance illustrate the interest in the use of non-specific herd productivity data for veterinary syndromic surveillance (31, 32). This study presents the comparison of three statistical methods to retrospectively detect the BTV-8 and SBV epidemics in the Netherlands based on routinely collected milk production records. The methods we chose to compare were directed at temporal abnormalities (Cusum), spatial abnormalities (Bayesian disease mapping) or a combination of both (spatiotemporal cluster analysis). Each of these methods has its own advantages and drawbacks (6, 9, 12, 33) but to our knowledge have not been applied simultaneously in a veterinary syndromic surveillance context for the early detection of emerging diseases.

### Baseline Model

Baseline models for milk production were constructed for three separate time periods using historical baselines of 52–104 weeks. A drawback of the use of time series is that a meaningful baseline can only be made when sufficient historical data is available. As milk production records from July 1, 2005 onwards were used, only 1 year of baseline was available when predicting milk production in the summer of 2006. Using only 1 year as baseline is suboptimal because abnormal weather conditions and, consequently, poor roughage quality and fluctuations in feed prices can have a large influence on the value of the baseline model. From the three time periods we examined, the BTV-model had the highest mean RMSE value, indicating the least fit of the baseline model. The SBV-model had the best fit of the baseline model. The Control-model had a mean RMSE value that was in between the BTV-model and SBV-model. This is supported by the distribution of residuals in the baseline period over all districts: the 5th percentile values of these distributions have a median value of −0.83 in the BTV-model, −0.68 in the SBV-model and −0.80 in the Control-model. From Figure 2 it is visible that the observed milk production in some weeks in the first half of 2009 showed outlying drops in milk yield. These were likely the result of extremely dry weather conditions resulting in poor grass yield (34). Each of the three methods picked up this drop in milk yield (results not shown), indicating the methods are robust to detect outlying observations. However, such non-disease related events have certainly influenced the fit of the baseline model for the SBV-model and the Control-model. Also, baseline periods including disease outbreaks should be used with caution. In the model we used to detect the re-emergence of BTV-8 in 2007, data from 2006 covering the initial outbreak period of BTV-8 was included in the baseline. This might have hampered detection of drops in milk production during the outbreak period in 2007. When implementing a syndromic surveillance system using predictions based on a historical baseline, it might be worthwhile to replace records from outbreak episodes or other abnormal events in a baseline by normalized historic observations. Due to privacy issues, milk yield levels per herd were aggregated by two-digit postal district. A disadvantage of this approach is that the administrative borders of a postal district do not reflect the spatial distribution of epidemiological factors, such as herd size and herd density (35). Variation in herd density between postal districts was accounted for by including the number of herds per postal district in the statistical models. Alternatively, one could aggregate data by a grid size that ensures each grid to represent an equally large proportion of the population at risk, provided that geographical coordinates of the unit of interest are known.

### Evaluation of Performance

In this study we used authentic data from years with emerging disease outbreaks, varying in magnitude and duration, and from years without major disease outbreaks. The use of authentic data in combination with the prospective nature of the methods we applied provided the possibility of illustrating which output (alerts) will be generated in reality if the methods were to be applied in real-time. This allows evaluation of the true effectiveness of the detection algorithms. A disadvantage of the use of authentic data for evaluation of outbreak detection methods is the difficulty of defining which alert is the result of an outbreak and which is not. In addition, in order to calculate performance metrics, such as sensitivity, timeliness and specificity, a gold standard is essential, such as the location and time of introduction of the pathogen responsible for the disease outbreak. In this study we used clinical suspicions in cattle as reference signals to define the start of the BTV-8 and SBV outbreaks per district. It was a challenge to classify the generated alerts as “true” or “false,” as the actual time between introduction of these viruses and detection of introduction was unknown. Therefore, the value of alerts generated prior to the first notifications of suspected cases remained unclear. Also, voluntary notifications of clinical suspicions might not be independent as the start of a disease outbreak is often communicated by the media, potentially influencing the number of notifications being made thereafter. In addition, disease awareness in years with major disease outbreaks will increase awareness in subsequent years. One way to overcome these issues is to evaluate algorithm performance using wholly simulated data or by simulating outbreaks of varying magnitude on datasets with a baseline based on authentic data (36).

### Sensitivity to Detect BTV-8 and SBV

In August 2006, BTV-8 appeared unexpectedly in northern Europe affecting parts of the Netherlands, Belgium, Germany and northern France (37). After successful overwintering in the region, BTV subsequently re-emerged in 2007 in all countries affected, in a far more extensive way than the first “wave” of the epidemic in 2006. BTV-8 is known to affect milk production in cattle, yet milk loss is not a prominent clinical sign in cattle (30). It is likely however that milk loss is initiated during the acute phase of the disease, but remains unnoticed in individual cattle several weeks before becoming clearly significant (38) or even completely unnoticed when only few cows are affected. The Bayesian disease mapping method generated two alerts in the early summer of 2006, 1–2 weeks before the first notifications of BTV-8 suspicions were made (39). The relatively limited impact of BTV-8 on milk production (18) in the Netherlands in 2006 and the aforementioned reduced quality of the baseline model might have contributed to the fact that only few alerts were generated in the summer of 2006. Alerts of decreased milk production, long before first clinical suspicions were made, were observed in a greater extent in 2007 in the districts whose notifications were the first signal of BTV-8's re-emergence. The degree of reduction in milk yield in spatiotemporal clusters—expressed as the difference between observed and predicted milk yield - was also higher in 2007 (on average −1.6 kg per cow) compared to 2006 (on average −1.2 kg per cow) (data not shown). The sequence of alerts in the neighboring southern districts clearly indicated a pattern of decreased milk production from the end of May to early July 2007. We classified a part of those alerts as false alerts as they were generated more than 4 weeks before first clinical suspicions were notified. However, it cannot be excluded that these alerts were the actual first signs of BTV-8's re-emergence. Evidently, increasing the length of the window to classify alerts as true or false, for example from 4 to 6 weeks before the reference signal, slightly improved sensitivity of detection of BTV-8 in 2007 by the methods, but not for 2006 (results not shown).

Spatiotemporal cluster analyses resulted in the most efficient detection of SBV, i.e., a high sensitivity was achieved with few alerts. The degree of reduction in milk yield in spatiotemporal clusters was on average −1.1 kg per cow in 2011 (results not shown). Irrespective of method used, the sensitivity of detection was higher for SBV than for BTV-8. This could have two reasons. Firstly, the trigger for reference signals we used for SBV was the same as the trigger for the outbreak alerts, i.e., for SBV they were both based on drops in milk production whereas the BTV-8 reference signals were based on more specific clinical signs. Secondly, as suggested earlier by Madouasse et al. (40), analysis of milk production data may produce an alert even when the impact of the disease on milk production is limited, provided that the disease spreads fast (as was the case with SBV, but not with BTV-8). More importantly, each method generated multiple alerts in 2011, of which the majority in August and September. This is in agreement with the time at which notifications of clinical suspicions in adult cattle, later confirmed as being SBV, were made by veterinarians from a number of districts. As it is known that SBV spread widely throughout the Netherlands within several months (or even weeks) in 2011/2012 (22) it is evident that clinical signs in cattle during the acute phase of the disease were not observed or not notified in the remaining districts (notifying suspicions was not mandatory up till December 2011). Therefore, the number of generated alerts in the summer of 2011 in the absence of reported clinical suspicions does suggest that, for each method, sensitivity of detecting SBV may be underestimated in this study. Yet, whether these alerts were the actual first signals of the SBV epidemic remains unknown.

### Specificity and Parameter Settings

Residual milk yield, not explained by seasonal fluctuations and trend in time, was used in this study as input for the outbreak detection methods. It is likely that a large part of the unexplained variability in milk production is caused by factors, such as climate, feed quality and feed price. In earlier work we suggested that specificity of outbreak detection methods based on milk production data might be improved if underlying regression models were to be extended with variables explaining climatological factors (17). Therefore, in this study, we added weekly mean ambient temperature and amount of rainfall for each district, based on its nearest weather station (41), to the baseline models. These factors did not alter the results considerably; the generated alerts differed only marginally and the mean RMSE per model was equal (results not shown).

Spatiotemporal cluster analysis was the only method that reached 100% specificity. Perhaps it is the evaluation of significance of only the most likely cluster by the space-time scan statistic that leads to such high specificity of this method. Nevertheless, specificity in 2010 was also high for the Cusum algorithm and Bayesian disease mapping analysis. The influence of parameter settings (such as the detection threshold) and the trade-off between sensitivity and specificity was clearly visible in 2006, 2007, and 2011. High absolute values of sensitivity were achieved by “loosening” detection thresholds, yet with an increase in total number of (false) alerts per year and decrease in predictive value as a—potentially expensive—consequence. The choice of parameter settings is therefore essential in each outbreak detection method. For the Cusum algorithm, this can be achieved by finding the most optimal value for the reference value *k* and control limit *h*. The choice of *k* could be based on a certain percentile of the frequency distribution or any other acceptable level of the parameter of interest (e.g., residuals or case counts). The choice of *h* however depends on the desired timeliness and specificity of the system, as it determines which deviations from *k* will lead to an alert. SaTScan offers a few calibration options in the Normal model we used, like setting the maximum spatial cluster size and the maximum temporal cluster size. The output was not sensitive to changes in the maximum temporal size (varied to 1, 2, and 4 weeks; results not shown). The most evident calibration possibilities in Bayesian disease mapping models, such as the one we used are the choice of the posterior exceedance probability threshold [for example P(res < −5)] and the desired confidence level to identify areas were the residual value (or relative risk) is below the detection limit (for example 95 or 99%). Other aspects which should be considered with care are the choice of prior distributions and which variance components to include in the model (correlated and uncorrelated). In general, the desired performance and purpose of the syndromic surveillance system should be leading the choice of parameter settings. Systems aiming at early detection of emerging diseases at all cost (i.e., false positive alerts are allowed) will aim for high sensitivity, whereas systems in which false positive alerts have large consequences (such as a trade ban), or follow-up costs are high, should aim for high specificity.

### Comparison of Methods

From the three methods we applied in this study, the Cusum algorithm was the most straightforward to construct and use. This feature and the fact that process behavior is examined chronologically and displayed in a graphical comprehensive manner are particular advantages of quality control charts, such as the Cusum algorithm (34). We applied the Cusum algorithm to 90 postal districts, monitoring each of these areas individually. A drawback of this approach is that if an outbreak occurs on the border between areas or only in a small part of an area, an important outbreak may be missed because it did not follow to the predefined geographical boundaries (10). Spatiotemporal cluster analysis using the space-time scan statistic does not take into account geographical boundaries, but imposes assumptions about the shape of disease patterns by finding clusters in the form of a circle or cylinder (36). The fact that the spatiotemporal cluster analysis of milk production performed best in our comparison could be due to the simultaneous assessment of spatial and temporal variation in milk yield, as opposed to the temporal Cusum algorithm and the spatial Bayesian disease mapping model. The main characteristic of Bayesian disease mapping models based on conditional autoregression (CAR) is to provide some shrinkage and spatial smoothing of raw relative risk estimates, resulting in a low sensitivity for detecting areas that only have a small excess risk (25). Strong local smoothing across neighboring areas, in particular smoothing of abrupt changes in relative risks is undesirable in a disease outbreak detection context (12). Bayesian disease mapping is however considered an appropriate tool for small area disease mapping (6). Compared to clinical surveillance, the Bayesian disease mapping model performed poorest out of the three methods we assessed, suggesting a need for a revision of the parameter settings used or an extension of the model to space and time.

From the methods we compared, the spatiotemporal approach provided in SaTScan's scan statistic has the greatest potential to be used for syndromic surveillance on milk production records. However, as suggested earlier (17), the added value of any syndromic surveillance system depends on several factors, such as the availability of demographic coverage of suitable data and the costs associated with the follow-up of alerts. Also, differences in the impact (on e.g., milk yield) and spread of an emerging disease might lead to different performance of the methods we proposed in this study. It is expected that the performance of the methods that were assessed in this study will be different when used for detection of diseases that are less clustered in space like vector-borne diseases generally are. For example, if movement of infectious animals results in simultaneous disease outbreaks in multiple non-adjacent areas, temporal methods are probably more suitable for early detection than spatial or spatiotemporal methods (as the indicator will be affected both inside and outside the cluster being tested). It would therefore be interesting to assess the performance of the methods under alternative disease pattern scenarios, for example that of directly transmitted diseases.

## Conclusion

When applied on routinely collected milk production data, spatiotemporal cluster analysis using the space-time scan statistic performed better than a temporal Cusum algorithm and a spatial Bayesian disease mapping model for early-detection of BTV-8 and SBV in cattle in the Netherlands. Compared to clinical surveillance, sensitivity and predictive alert values were high to detect SBV and low to detect BTV-8. Particularly in the years in which BTV-8 emerged and re-emerged, alerts of reduced milk production were generated long before first clinical suspicions were reported. It remains unknown whether these alerts represent the actual first signs of BTV-8 infections in cattle in the Netherlands.

## Data Availability Statement

The datasets generated for this study will not be made publicly available as the data has been provided by a commercial dairy processor, who wishes not to make their data publically available. Requests to access these datasets should be directed to (Dr. Veldhuis, a.veldhuis@gdanimalhealth.com).

## Author Contributions

AV, WS, HB-M, and GS contributed to the conception and design of the study. AV, WS, and HB-M developed the statistical models. AV performed the statistical analyses and wrote the first draft of the manuscript. All authors contributed to interpretation of the results, contributed to manuscript revision, read and approved the final version.

### Conflict of Interest

AV, WS, HB-M, MM, and GS are employed by company Royal GD. The remaining author declares that the research was conducted in the absence of any commercial or financial relationships that could be construed as a potential conflict of interest.
