# Supplementary material for: The Comparison of Three Statistical Models for Syndromic Surveillance in Cattle Using Milk Production Data
Source: Front Vet Sci. 2020 Mar 6;7:67. doi: 10.3389/fvets.2020.00067 (PMC7068209; doi:10.3389/fvets.2020.00067)
Supplement: Supplementary file 1 [file Data_Sheet_1.PDF]

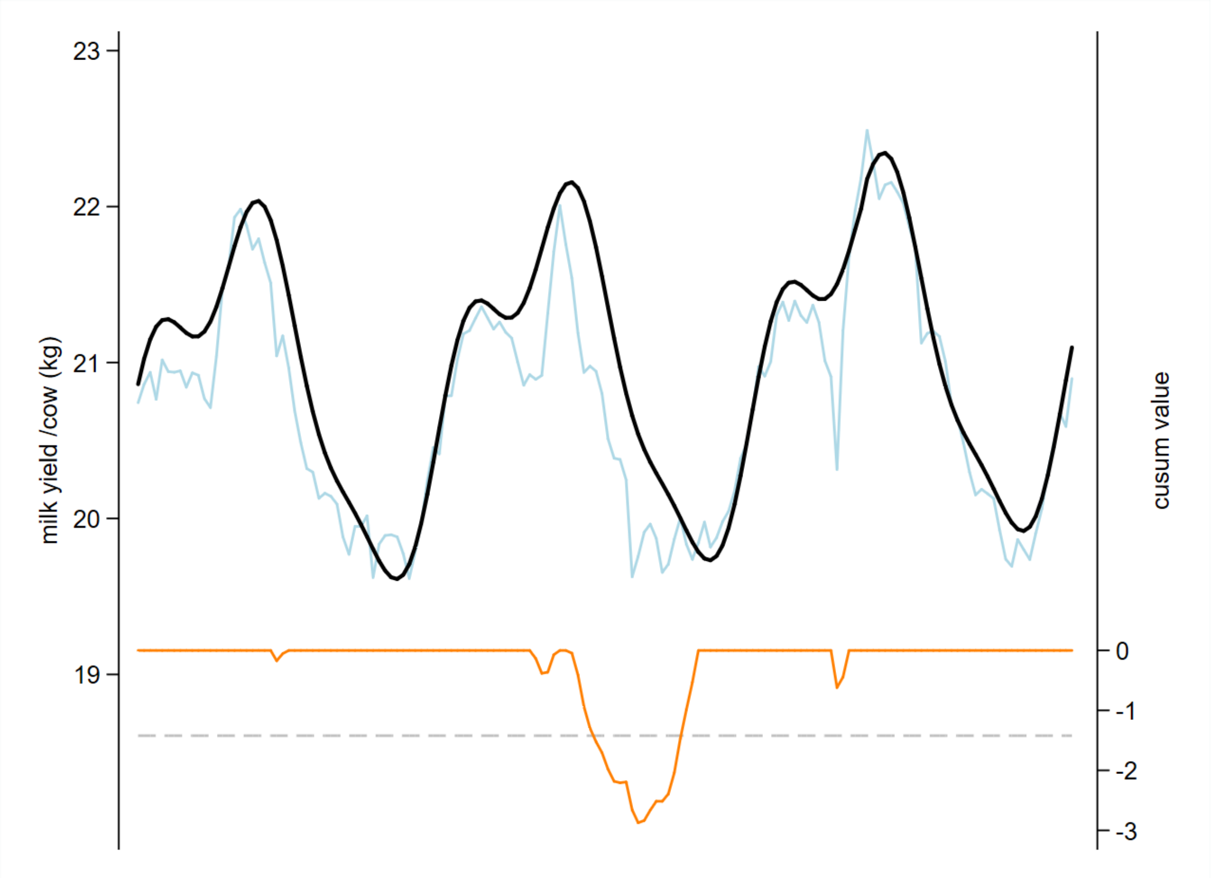

Supplementary Figure 1. Graphical example of a Cusum chart for a randomly chosen district, with mean observed milk yield per cow (blue), predicted milk yield per cow (black), the Cusum value (orange) and control limit *h* (dashed grey).
